# A Hospital Treasurer's Suggestion

**Published:** 1920-07-17

**Authors:** 


### The Hospital

The Workers' Newspaper of Administrative Medicine and Institutional Life, Administration, National Insurance and Health.

No. 1780, Vol. LXVIII. SATURDAY, JULY 17, 1920. PRICE TWOPENCE

### A HOSPITAL TREASURER'S SUGGESTION.

We publish this week an inspiring address by Mr. Leonard Keyser, honorary treasurer of the Royal Hampshire County Hospital at Winchester. It is well worth study and contains more than one suggestion which should appeal to the public spirit of the trades unionists who listened to it. Mr. Keyser is beginning a stiff task. He wants every worker to contribute a fixed weekly sum to the hospital in order to persuade the richer men of the district to pay off the overdraft of £6,000, which is bound to recur unless the future income is maintained at a higher figure than the present. Mr. Keyser, like other hospital treasurers, finds that the wealthy are eager to help the workers through the hospital when the latter are sick, if the workers when they are well will maintain the hospital properly. For each class has its own duty to the victims of ill-health. The rich, who benefit by the doctors' training in hospitals but do not go to hospitals themselves, provided in the past the large capital sums needed to create the institution and to provide it with a small endowment. It is for the workers who use the hospitals in sickness to help one another by subscribing when they are well for the benefit of their brothers in the wards. Mr. Keyser further invites them to do away with the weekly collecting-boxes, by which the generous give more and others less than their fair share, and to substitute a card system of collecting. He also, we understand, would like to see this card used to regularise the treatment for the benefit of those who can produce the card. The card would prove that its owner was a regular subscriber to his local hospital, and therefore eligible for free treatment there. But the plan could be developed further. If all the hospitals adopted the card system, Mr. Keyser urges that a wage-earner, who subscribed to his local hospital, could be transferred to another whenever he happened to move. Suppose a man to be a subscriber to the Royal Sussex Hospital, if his work carried him to Reading he would then, on Mr. Keyser's plan, be eligible for the treatment at the Royal County Hospital there.

We would ask if this scheme, which is simple and well reasoned, could not be carried a step further. Supposing the card system generally adopted, why should not its owner be eligible for treatment at any other hospital within reasonable distance where there happened to be an earlier vacancy? This step toward co-ordination could be taken in conjunction with the regional plan lately welcomed by the British Hospitals Association, which could have in each of its twelve centres a sorting-office to deal with transfers of the kind. An alternative would be for the new county organisations under the Red Cross to arrange the matter, which would be an addition to the scheme already sketched by Mr. Keyser.

At all events, he has set a good example by taking the trade unionists into his counsels, and his speech shows a frankness and good sense to which anybody of intelligent men should respond. Another point which it makes is an old one which is sometimes missed in industrial areas. We mean his insistence on the fact that subscriptions from any class, and whether paid by the year or by the week, are subscriptions paid out of goodwill to *other people*, and carry with them no more right or claim than the victim had upon the Good Samaritan of the parable. It is sickness alone which gives a man a right to hospital treatment. It is health which brings the duty to give to others. We believe also that many large sums are lost to hospitals because would-be donors hold back, and they hold back because if their duty is to help the hospitals out of a difficulty, the duty of well-to-do workmen to maintain it is sometimes unfulfilled at the present time. Yet if rich and poor have different needs and claims, they have also different duties, and the position is only healthy when these are clearly defined. The need of the moment is for the methodical development of existing sources of income. That is why the card system is preferable and more dignified than the weekly collecting-box.

The plan outlined above deserves notice, because that which is being attempted in Hampshire needs to be done wherever it has not been properly tried.

How slowly progress is made can be seen from the fact that Sheffield is only now debating the scheme for contributions from employers and employed already established in Leeds. In the letter which we publish from Sir Arthur Stanley, based on Sir Napier Burnett's survey, he urges similar points. If, he says, the burden of debt can be removed, he feels confident that "the subscriptions and donations of the public, which were sufficient to maintain the hospitals in pre-war days, will expand to meet the growing necessities." We think so, too. There is only one danger, and it comes from those hospitals which have conducted their finance in the past by personal appeals to all and sundry, and when these fail have no other resources, except to clamour for Government support. That is why each hospital intent on a methodical organisation of subscriptions from employers and employed sets an example which will be doubly valuable if it induces other institutions to be equally thorough.

